# Dual-modality emergency management of a rare cause of an aorto-oesophageal fistula: a case report

**DOI:** 10.1093/jscr/rjab035

**Published:** 2021-03-08

**Authors:** Rishi Vasanthan, Yasmin Tabbakh, Sophie Doran, Daryll Baker, Julian Hague, Yassar Qureshi, Khaled Dawas, Borzoueh Mohammadi

**Affiliations:** Department of Head and Neck Surgery, University College London Hospitals NHS Foundation Trust, London, UK; Department of Upper Gastro-Intestinal Surgery, University College London Hospitals NHS Foundation Trust, London, UK; Department of Metabolism, Digestion & Reproduction, Hammersmith Hospital Clinical Research Facility, Imperial College London, London, UK; Department of Upper Gastro-Intestinal Surgery, University College London Hospitals NHS Foundation Trust, London, UK; Department of Vascular Surgery, Royal Free London NHS Foundation Trust, London, UK; Department of Vascular Surgery, University College London Hospitals NHS Foundation Trust, London, UK; Department of Radiology, University College London Hospitals NHS Foundation Trust, London, UK; Department of Radiology, Royal Free London NHS Foundation Trust, London, UK; Department of Upper Gastro-Intestinal Surgery, University College London Hospitals NHS Foundation Trust, London, UK; Department of Upper Gastro-Intestinal Surgery, University College London Hospitals NHS Foundation Trust, London, UK; Department of Upper Gastro-Intestinal Surgery, University College London Hospitals NHS Foundation Trust, London, UK

**Keywords:** General surgery, Aorta, Upper Gastrointestinal Tract, Oesophagus, Endovascular Procedures, interventional Radiology

## Abstract

Aorto-oesophageal fistula (AOF) is a life-threatening condition that usually presents with upper gastro-intestinal haemorrhage. This case report details the emergency presentation and management of a 51-year-old male who presented with hematemesis secondary to an impacted denture (ingested two years previously) in the oesophagus that had led to an AOF. This necessitated urgent thoracic endovascular aortic repair followed by thoracotomy, oesophagotomy, T-tube insertion and oesophagostomy. This is the first documentation in the literature of the dual-modality management for this rare cause of AOF and demonstrates the multidisciplinary approach to successful management of this complex yet rare presentation.

## INTRODUCTION

Aorto-oesophageal fistula (AOF) usually results in fatal upper gastro-intestinal (UGI) haemorrhage, despite developments in treatment [1]. Causes of AOF include thoracic aortic aneurysm, malignancy and foreign body ingestion. Since 1994, the advent of thoracic endovascular aortic repair (TEVAR) has revolutionized the condition’s management [2]. In-hospital mortality of AOF can be up to 42% without treatment and 28.2% after treatment with TEVAR [1].

We present a rare case of AOF secondary to a denture ingested two years earlier and describe the immediate surgical management. This cause has been documented only four times in the literature (three diagnosed postmortem) with the last case 20 years ago [3–6]. To the best of the authors’ knowledge, this is the first case that reports the dual-modality management of this unusual cause of AOF.

## CASE

A 51-year-old male was referred via the suspected cancer pathway with dysphagia and weight loss. An oesophago-gastro-duodenoscopy (OGD) revealed a dental bridge impacted at 20–25 cm. This appeared embedded in the oesophageal mucosa and was not removed due to risk of perforation. The patient reported that the denture had been there for two years. The case was planned for discussion at a multi-disciplinary team meeting to decide on the safest method of removal.

A month later, the patient presented to the Emergency Department of a peripheral hospital following 2 L of hematemesis. Associated symptoms included chest pain, fever and light-headedness. Past medical history included hemiparesis (after a road traffic collision requiring a craniotomy 30 years earlier). On arrival, his heart rate was 128 bpm, respiratory rate of 22 min^−1^, blood pressure of 131/65 mmHg, saturations of 94% on room air and he was apyrexial. He was given intravenous tranexamic acid, vitamin K, pantoprazole, tazocin and 2 units of packed red blood cells (pRBCs). Blood tests showed a haemoglobin of 150 g/L, white cell count of 24 (×10^7^/L), platelets of 503 (×10^7^/L), lactate of 2.2 mmol/L and normal clotting, renal and liver function tests.

**Figure 1:**
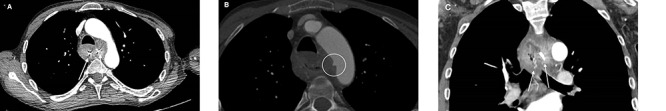
A computerized tomography scan with contrast in the arterial phase showing the AOF and denture in the oesophagus. (**A**) Axial plane view with arrows pointing to denture within the oesophagus, (**B**) axial plane view with AOF circled and (**C**) coronal plane view with arrows pointing to denture within the oesophagus.

An urgent CT scan with triple-phase contrast revealed an impacted foreign body in the mid-oesophagus, with peri-oesophageal fat-stranding, forming an inflammatory mass abutting the aortic arch. There was no evidence of perforation. There was subtle evidence of aortic arch ulceration (outpouching at the medial aspect of the aortic arch directed towards the oesophagus at the level of the foreign body) suggestive of an AOF, as shown in Fig. 1. There was no extravasation of contrast to indicate active bleeding, although we suspect he had suffered an initial herald bleed.

The patient had further hematemesis, becoming hemodynamically unstable with a tachycardia of 140 bpm. His haemoglobin dropped to 127 g/L and white cell count was 35.7 (×10^7^/L).

The patient was referred to the tertiary UGI centre and was blue-light transferred, intubated and ventilated, having received 6 units of pRBCs in total.

**Figure 2:**
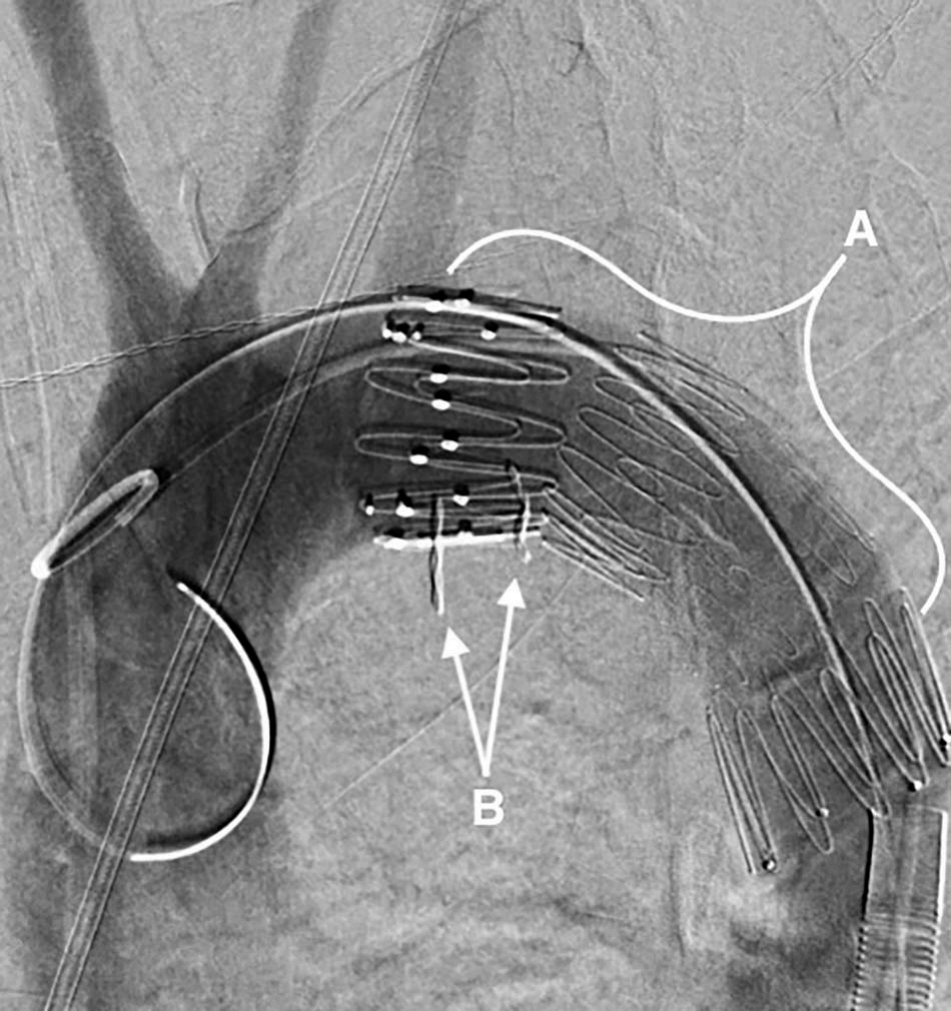
A fluoroscopy image in the sagittal plane showing: (**A**) the stent-graft within the aorta and (**B**) the denture.

On arrival, the patient underwent an urgent thoracic endovascular aortic repair (TEVAR). This was successfully performed with a Zenith TX2® (Cook Medical, Indiana, USA) 28 × 80 mm endovascular stent-graft deployed across the aortic ulceration with the left subclavian artery spared, as demonstrated in Fig. 2. Post-procedure he was admitted to the intensive care unit (ICU).

The following day, the patient was taken to theatre. An on-table OGD revealed a proximally dilated patulous mega-oesophagus, with the dental bridge unmoved from previously. This could not be retrieved endoscopically; therefore, a right postero-lateral thoracotomy was performed to access the oesophagus. A longitudinal oesophagotomy below the level of the foreign body allowed removal of the dental bridge in its entirety. During this time, with single lung ventilation ongoing, the patient became unstable from a respiratory perspective. Therefore, it was decided to close the oesophagotomy with a 3–0 polydioxanone suture around a 12 Fr T-tube, rather than perform an oesophageal resection. A 28 Fr chest drain was placed adjacent to the oesophagotomy.

To minimise further contamination from the fistula, with the aortic stent-graft now in situ, the decision was made to exclude the aorto-oesophageal inflammatory mass by forming a left-sided oesophagostomy and a covering Penrose drain. Neck dissection was complicated following chronic obstruction, with a loss of tissue plane due to severe inflammation. Next, an upper midline laparotomy allowed formation of a venting gastrostomy using an 18 Fr Foley catheter, to reduce gastro-oesophageal reflux into the native thoracic oesophagus in situ. A 14 Fr MIC surgical jejunostomy tube was also sited. The patient returned to ICU and was treated with broad-spectrum antibiotics and antifungals.

Day one post-operatively, the patient was successfully extubated, weaned off vasopressor support and started jejunal feed. He had a hoarse voice, and a left vocal cord palsy secondary to recurrent laryngeal nerve injury was diagnosed on flexible nasendoscopy. He was stepped down to the ward on day 3 and a day 9 CT showed unremarkable appearances of the TEVAR and no aortic extravasation of contrast. One month post-operatively, chest and neck drains were removed, with the T-tube left in situ for 8 weeks minimum. Subsequent management will involve thoracotomy, oesophageal resection, gastric conduit formation (mediastinal or retrosternal route) and cervical anastomosis.

## DISCUSSION

AOF is a rare and life-threatening condition. The management includes haemorrhage control and treatment of the diseased oesophagus. Most commonly the latter is in the form of a resection (with or without immediate reconstruction), primary fistula repair or oesophageal stenting [7–9].

Here, TEVAR was successfully used to control the initial haemorrhage; however, resection was not possible due to patient instability. This case highlights that repairing the oesophageal defect around a T-tube and performing a defunctioning oesophagostomy provides an alternative method to lessen the risk of ongoing sepsis, allowing a semi-elective reconstruction at a later date.

A seemingly benign denture can cause a significant inflammatory response with progression to AOF, advocating urgent removal if retained in the UGI tract. Therefore, foreign body and AOF are an important but rare cause of UGI bleeding to consider in select patients.

## References

[1] Li S, Gao F, Hu H-O, Shi J, Zhang J. Review article risk factors for mortality in patients with aortoesophageal fistula related to aortic lesions. Gastroenterology Research and Practice 2020;2020:4850287. doi: 10.1155/2020/4850287. PMC7519457 33014040

[2] Dake MD, Miller DC, Semba CP, Mitchell RS, Walker PJ, Liddell RP. Transluminal placement of endovascular stent-grafts for the treatment of descending thoracic aortic aneurysms. N Engl J Med 1994;331:1729–34. 7984192 10.1056/NEJM199412293312601

[3] Taha AS, Nakshabendi I, Russell RI. Vocal cord paralysis and oesophago-broncho-aortic fistula complicating foreign body-induced oesophageal perforation. Postgrad Med J 1992;68:277–8. 10.1136/pgmj.68.798.277 PMC2399283 1409192

[4] Singh B, Puri ND, Kakar PK. A fatal denture in the oesophagus. J Laryngol Otol 1978;92:829–31. 10.1017/s0022215100086175 359739

[5] Fujita H, Noda T, Hatakeyama T, Nameki H, Otsuka M, Nishida K. Perforation of the aorta induced by a swallowed denture in the esophagus: a case report and the review of the literatures. J Japanese Assoc Thorac Surg 1977;25:1490–6. 609015

[6] Pohlmann JT, Thomsen NO. Aortoesophageal fistula. A rare cause of hematemesis. Ugeskr Laeger 1996;158:4772–4. 8801688

[7] Aday U, Çetin DA, Çiyiltepe H, Gündeş E, Bozdağ E, Senger AS. Cause of mortality in aortoesophageal fistula: oesophageal sepsis. A case report. Prz Gastroenterol 2017;12:222–5. 29123585 10.5114/pg.2017.70476 PMC5672711

[8] Göbölös L, Miskolczi S, Pousios D, Tsang GM, Livesey SA, Barlow CW, et al. Management options for aorto-oesophageal fistula: case histories and review of the literature. Perfus (United Kingdom) 2013;28:286–90. 10.1177/0267659113476329 23401340

[9] Omura A, Yoshida M, Koda Y, Mukohara N. Surgical management without resection of the oesophagus for aorto-oesophageal fistula secondary to aortic arch aneurysm rupture. Interact Cardiovasc Thorac Surg 2016;23:985–7. 27543651 10.1093/icvts/ivw239

